# An All-in-One Solid State Thin-Layer Potentiometric Sensor and Biosensor Based on Three-Dimensional Origami Paper Microfluidics

**DOI:** 10.3390/bios11020044

**Published:** 2021-02-10

**Authors:** Shiva Pesaran, Elmira Rafatmah, Bahram Hemmateenejad

**Affiliations:** Chemistry Department, Shiraz University, Shiraz 71454, Iran; sh.pesaran@shirazu.ac.ir (S.P.); elmirarafatmah@shirazu.ac.ir (E.R.)

**Keywords:** paper-based origami sensor, three-dimensional microfluidic, potentiometric, biosensor, carbon paste electrode

## Abstract

An origami three-dimensional design of a paper-based potentiometric sensor is described. In its simplest form, this electrochemical paper-based analytical device (ePAD) is made from three small parts of the paper. Paper layers are folded on each other for the integration of a solid contact ion selective electrode (here a carbon-paste composite electrode) and a solid-state pseudo-reference electrode (here writing pencil 6B on the paper), which are in contact with a hydrophilic channel fabricated on the middle part (third part) of the paper. In this case, the pseudo-reference and working electrodes are connected to the two sides of the hydrophilic channel and hence the distance between them is as low as the width of paper. The unmodified carbon paste electrode (UCPE) and modification with the crown ether benzo15-crown-5 (B15C5) represented a very high sensitivity to Cu (II) and Cd^2+^ ions, respectively. The sensor responded to H_2_O_2_ using MnO_2_-doped carbon paste electrode (CPE). Furthermore, a biosensor was achieved by the addition of glucose oxidase to the MnO_2_-doped CPE and hence made it selective to glucose with ultra-sensitivity. In addition to very high sensitivity, our device benefits from consuming a very low volume of sample (10.0 µL) and automatic sampling without need for sampling devices.

## 1. Introduction

Electroanalytical sensors based on ion-selective potentiometry have gained a lot of interests in analytical chemistry because of their simplicity, high speed, low cost, wide dynamic range and potential for miniaturization. Potentiometry is still the universal approach for pH measurement and is among significant measurement techniques in clinical laboratories. Now-a-day, ion-selective electrodes (ISE) have also become versatile tools for the detection and analysis of organic and inorganic species [1]. In addition, the development of thin-layer potentiometry and solid contact ion-selective electrodes (SCISE) during the last few decades provide potentiometric sensors as an ideal candidate for fabrication of miniaturized sensors with the enhanced sensitivity and simplified construction and operation methods [2,3].

In spite of the drastic properties encountered for the potentiometric ion-selective sensors, further, improvements are required. Fabrication of sensors having a lower cost (for large scale applications and using them as a point of care diagnostic tools), higher sensitivity and diminished memory effects, being easy to prepare and operate and of course being portable are appreciated. In this regard, paper-based analytical devices (PAD) have becoming attractive platforms for the fabrication of novel and affordable sensors ad biosensors [4].

Paper substrate as micro-PAD (µPAD) in the form of microfluidic was first reported in 2007 by Whitesides et al. [5]. Paper as a substrate has recently attracted much attention because it is a flexible, readily available with low cost and is biocompatible [6,7]. The µPADs, which rely on the transportation of solutions by capillary force through a device [8], are fabricated and patterned into one-dimensional (1D), two-dimensional (2D) and more complex three-dimensional (3D) microfluidic devices [9,10]. In the 3D devices, which allow rapid distribution of the sample in the z-direction [11], the origami design can effectively eliminate problems of reagent diffusion by lateral flow in the channels of planar paper devices [12]. 

Since a few years ago, resourceful µPADs have been developed with a variety of detection methods including but not limited to colorimetry [13,14,15], fluorescence [16], chemiluminescence [17], electrochemistry [18,19] and electroluminescence [20]. Among the colorimetric and electrochemical methods, which have found more extensive applications, paper-based electrochemical devices (ePAD), which were firstly published by Dungchai [21], have the advantages of insensitivity to color interferences and fluctuation in the environmental light and are being more quantitative with higher sensitivity and wider linear range compared to colorimetric-based PADs.

According to the explanations outlined above, we decided to design an origami paper-based potentiometric sensor applicable in sensing of a wide range of species (both chemical and biochemical). Monitoring of metal ion concentration is very important for prevention of negative impacts that it can have on human health. The performance of the proposed µPAD was first investigated in analysis of inorganic ions such as Cu^2+^, Cr^3+^, Ag^+^ and Hg^2+^. Chromium can cause allergic reactions on the skin and can be carcinogenic, mercury compounds are very strong poisons, copper is necessary for different chemical and bio-chemical processes in the body, but it can be toxic above a certain concentration. After-wards it was decided to assess this origami paper-based potentiometric sensor’s application in detecting of glucose as bioanalyte. It was observed that the origami structure of the sensor showed its significance by comparing the obtained detection limits (DLs) with previous similar reported bulk methods.

## 2. Materials and Methods

### 2.1. Materials and Equipment

D-Fructose, sucrose, D-maltose, D-galactose, lactose, glycogen, graphite powder, MnO_2_, KMnO_4_, Na_2_C_2_O_4_, NH_4_Cl, Cd(NO_3_)_2_·4H_2_O, K_2_HPO_4_, Nujol oil, benzo15-crown-5, Fe(NO_3_)_3_·9H_2_O, Ni(NO_3_)_2_·6H_2_O, KNO_3_, sodium acetate, urea, uric acid, NaCl, KCl, CaCl_2_·2H_2_O, MgSO_4_·7H_2_O, NaHCO_3_, Na_2_SO_4_, Na_2_HPO_4_, NaH_2_PO_4_·H_2_O, ammonia solution 25%, hydrogen peroxide 30% and murexide were all purchased from Merck. KH_2_PO_4_, creatinine, Cu(NO_3_)_2_•3H_2_O, AgNO_3_, Hg(NO_3_)_2_·H_2_O, [Cr(H_2_O)_6_]NO_3_)_3_·3H_2_O were purchased from Fluka. L(+)-Cysteine and Glucose oxidase (GOx), from Aspergillus niger type II, were purchased from Riedel-de-Haën and Sigma-Aldrich respectively. Na_3_C_6_H_5_O_7_·2H_2_O, D-glucose and glacial acetic acid were purchased from BDH Chemicals while Schleicher & Schuell^®^ (s&s) Grade 2040b qualitative filter paper (with a thickness of is 0.2 mm) was used for device fabrication. Carbon nanotube (CNT) was a gift of Dr. Doroodmand’s lab. Koh-i-Noor Hardtmuth pencils 3B, 4B, 6B, 9B, 4H and HB of different commercial brands were collected from local stores. All solutions were prepared using double deionized water.

An HP LaserJet 1320 printer from HP was used to print the devices. A Memmert Incubator Oven INB200 was used for curing the printed μPADs. Electrochemical measurements were made using an AZ-86502 bench top pH meter at room temperature (25.0 °C), Bionime GM110 Blood glucose monitor was used as reference method. A lab-made potentiometer was used for wifi sending of potential data on a mobile phone. The scanning electron micrographs (SEM) were obtained with a TESCAN model VEGA3 instrument.

### 2.2. Device Fabrication

**Figure 1:**
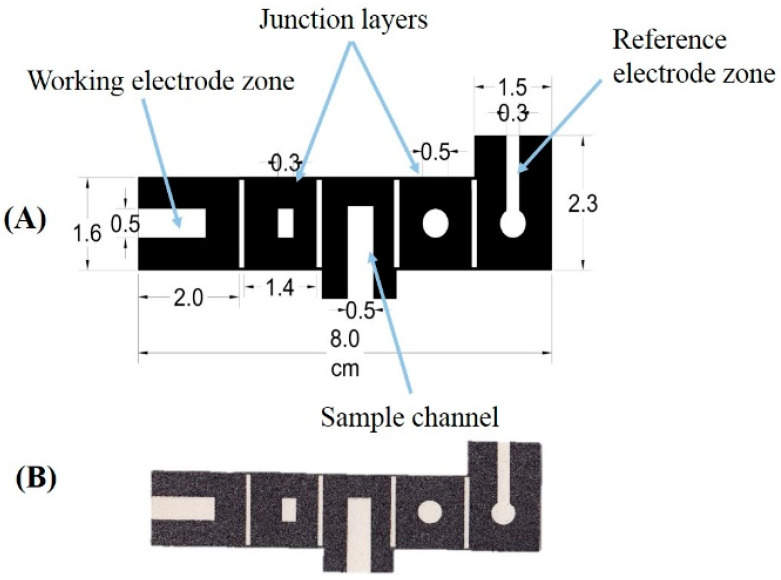
(**A**) Schematic diagram for fabrication of origami potentiometric device pattern. (**B**) The image of fabricated origami potentiometric device. The numbers denote the sizes in cm.

The designed pattern in AutoCAD software was laser printed on one side of the filter paper. Precision sensor pattern details are presented in Figure 1, where white and black colors represent hydrophilic and hydrophobic area of the sensor, respectively. As shown in Figure 1, in the left and right layers are provided hydrophilic zones for working and reference electrodes, respectively. The hydrophilic zone in the middle layer is used for sampling and plays the role of sample vessel. The device includes two other layers with small hydrophilic area, which are used as channels for connecting electrodes with sample solution. The size of sensor after folding it in the origami shape is around (2 × 2 cm). Hydrophobization of the printed areas was performed by heating them in the oven with a temperature of 200.0 °C for an hour [22].

Indicator electrodes were fabricated by pressing the carbon paste mixture through an iron mold attached to the paper with the aim of a magnet. A uniform carbon paste was prepared by mixing an appropriate amount of graphite powder, Nujol oil, and modifier for 20.0 to 25.0 min. Finally, polishing the pressed electrode surface on an oily paper was performed until a compact and smooth electrode was obtained. Pseudo-reference electrodes were simply prepared by drawing a pencil on the filter paper. Six types of pencils (9B, 6B, 4B, 3B, 4H, HB) with various carbon content were used.

### 2.3. Potentiometric Measurements Using the ePAD

**Scheme 1:**
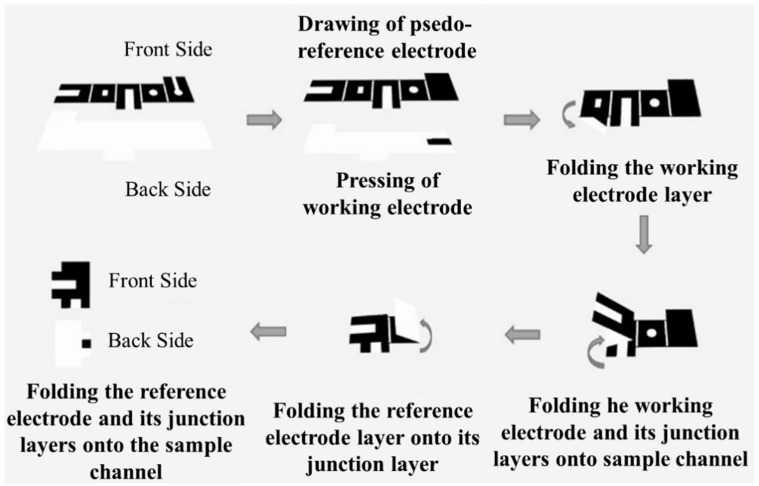
Schematic representation of the folding processes for a three-dimensional origami paper-based potentiometric device.

Folding of the sensor (Scheme 1) was performed by first bending the indicator electrode layer counterclockwise. Then the two layers of the indicator electrode and its square hydrophilic junction layer were folded on the sample channel. The last two layers of the pseudo-reference electrode and its circular hydrophilic layer were first folded on each other and then they were bent to the back side of the sample channel.

After folding the prepared sensor, 50.0 µL of sample solution was loaded to the sample channel. This was done by placing the beginning of the sample channel in the solution container. By capillary effect, sample solution moves through the proposed channel in the folded sensor. The sample volume was set to be as much as the channel becomes saturated from the sample. The device was then sandwiched among two glass sheets using clothespins. In order to increase the repeatability, potentiometer connection was stabilized by fixing the potentiometer alligators and the proposed sensor (Figure S1). Alligators were fixed on the transparent Flexi sheet (150.0 × 150.0 mm and thickness of 5.0 mm) with the aim of fast adhesive at a 90-degree angle toward each other. Then the sensor was kept stable in its situation by a small clamp (a wooden clothespin). The potential difference among the indicator and the pseudo-reference electrodes was measured afterward.

### 2.4. Inorganic Ions Potentiometric Measurement

The basis of the working electrode in this assay is carbon-paste electrode, which has represented selectivity toward potentiometric sensing of some metal ions [23]. So, we firstly applied our device for measurement of metal ions including Cu^2+^, Cr^3+^, Ag^+^, and Hg^2+^ by carbon paste (72 wt % of graphite powder and 28 wt % of Nujol oil) indicator electrodes and benzo15-crown-5 (B15C5) modified electrode (71% graphite, 25% Nujol oil and 4% B15C5) for measurement of Cd^2+^. The CPE was prepared by hand mixing of pure graphite powder (0.1 g) and Nojul oil (0.04 g), which were placed in a mortar and mixed well for 20–25 min. Moreover, modified CPE with B15C5 were prepared by hand mixing of pure graphite powder (0.1 g), B15C5 (5.6 × 10^−3^ g) and Nojul oil (0.03 g), placed in a mortar and mixed well for 20–25 min to form a uniform paste. Therefore, indicator electrodes were supposed to be fabricated by pressing the carbon paste mixture through an iron mold attached to the paper with the aim of a magnet, followed by polishing the pressed electrode surface on an oily paper. On the other hand, the reference electrode was fabricated using a 6B pencil to fill the designated area. In addition to electrode preparation, ionic strength and the pH were adjusted by capillary movement of KNO_3_ 0.1 mol L^−1^ in HAc-NaAc buffer 0.1 mol L^−1^ pH 5.0 in the sample channel. After dryness of the sensor in room temperature for 4.0 min, the sensor was folded in the right manner so it was ready for ion determination. The bottom side of sample channel was placed in the analyte solution so the sample would rise by capillary effect to the top of the channel. By attachment of potentiometer alligators to the sensor electrodes, potential difference related to the ion concentration was read.

### 2.5. Measurement of H_2_O_2_ and Glucose

The modified CPE with MnO_2_ was prepared by hand mixing well of pure graphite powder (0.1 g), MnO_2_ (5.6 × 10^−3^ g) and Nojul oil (0.03 g) in a mortar for 20–25 min to form a uniform paste. The other procedure was like that explained for sensing of metal ions. However, here, ionic strength and the pH were adjusted by capillary movement of NH_3_-NH_4_Cl buffer solution 0.1 mol L^−1^ with pH 8.5 in the sample channel.

### 2.6. Measurement of Glucose

The process of preparation the glucose sensor is similar to the hydrogen peroxide sensor, with the difference that, 2.5 μL of GO_x_ solution (0.1 g of GO_x_ in 100.0 μL of 0.1 mol·L^−1^ phosphate buffer) was dropped cast on the bottom of the indicator electrode before introduction of sample solution.

### 2.7. Real Sample

Four different blood samples were gathered and diluted by a saline solution with a 1:1000 ratio. Dilution was done to decrease the sample glucose concentration such that it would be in the linear range of the sensor. The related EMF of each sample was measured by directly loading of blood to the ePAD without any pre-separation step.

## 3. Results and Discussion

### 3.1. Sensor Design

The main objective of this design to decrease the distance between the indicator and the pseudo-reference electrodes which would improve the detection limit [24]. As a result, a three-dimensional origami device was considered. The device contains 5 paper layers (Figure 1). The first and the last ones are the electrode layers while the middle layer includes the sample channel. The other two layers contain connecting channels. It should be mentioned that the carbon paste indicator electrode had been placed at the backside of the sensor while the pseudo-reference electrode was drawn by pencil on the side which the deigned pattern had been printed.

For optimization of ePAD geometry and investigation of some analytical features of the sensor, unmodified carbon paste electrode (UCPE) was used as an indicator electrode. In bulk solution, UCPE represented selectivity toward metal ions such as Cu^2+^, Ag^+^, Cr^3+^ and Hg^2+^ [23,25]. Here, in the preliminary investigations, the response of ePAD to Cu^2+^ ion was followed.

In the preliminary investigation, it was found the width of sampling channel effected significantly the precision. In order to find the optimum dimension, four different sample channel patterns were designed and compared (Figure S2): (A) a sensor without sample channel (all part of the middle layer was used as sampling pad); (B) using a sample channel which is wider than electrode width; (C) channel width equal to the electrodes’ width and (D) the sample channel thinner than the electrodes’ width. The responses of the ePADs to 1.0 × 10^−6^ mol·L^−1^ solution of Cu^2+^ were investigated for 3 replicate measurements, and the relative standard deviation was calculated.

When the width of sampling channel was considered thinner than the width of electrodes, the signal was totally unstable and it was always fluctuating. By increasing the width of the sampling channel to the same size of the electrodes, the most stable results (relative standard deviation of 2.8%) were obtained. However, the signal instability was increased again by increasing the width of sampling channel. For the design, in which no sample channel was fabricated, smaller signals and less stable were achieved. Thus, in the future studies, the width of the sample channel was considered as the same size of electrode width. As shown in Supplementary Materials Figure S3, 50.0 μL of sample solution is required to fully fill the sampling channel.

In the next step, the number of junction layers was examined to see if it had any effect on the results. It has been reported that in the origami designs, increasing the number of layers may increase the quality of measurements by increasing the uniformity of analyte distribution [26]. In this part of study, 3-point calibration curves were plotted for each design and the slope of calibration curve and the goodness of fit (R^2^) were used as decision criteria. In the first design (3-layer design), the electrodes are in direct contact with two different sides of the sample pad. Hence, the distance between the electrodes is as the width of the filter paper, which is around 0.23 mm. For this design, a super-Nernstian slope (as is expected for Cu^2+^ ion) [27] with acceptable correlation coefficient was obtained. However, better results were achieved by adding one layer between the electrodes and the sampling pad (5-layer design). The super-Nernstian slope was not affected significantly. However, the quality of fitting, which was the results of better reproducibility was increased from R^2^ = 0.90 to R^2^ = 0.97. By adding one more layer to both sides (7-layer design), the quality of fit remained the same, but the slope was decreased significantly. By increasing the distance between the electrodes, the thin-layer condition may break. Moreover, the analyte may not reach to the electrodes in the same concentration as in the sample tube. As we reported previously, silver ions can be coordinated with hydroxy groups of paper cellulose [25] and this more likely to happen for copper ions too.

### 3.2. Selection of Suitable Pencil for Pseudo-Reference Electrode

Graphite pencils are attractive as an electrode material for paper-based analytical devices [28]. Pencils are cheap, highly conductive and available in many different diameters and lengths [29]. Therefore, the pseudo-reference electrode of the designed three-dimensional ePAD was simply prepared by drawing a graphite pencil on the paper substrate [30]. To find an appropriate pencil for this purpose, various sensors were prepared using 9B, 6B, 4B, 3B, 4H, HB pencils (B-grade have a higher carbon content while H-grade pencil contains more clay) as a pseudo-reference electrode. Obtained calibration curves for Cu^2+^ determination in Figure S6, showed that 6B and 4B pencils would result in a calibration curve with higher sensitivity and better correlation coefficients (R^2^). The 9B pencil would cause in a thick graphite network on the paper surface. This graphitic layer is weakly bonded by van der Waals force which its surface condition can be easily affected by the addition of a defined sample solution. On the other hand, the pseudo-reference electrode prepared by H pencils would contain a large amount of clay. This would increase the electrode hardness which also leads to a brittle and fragile electrode [31]. The surface morphology of the electrodes was investigated by SEM (Figure S7). Pseudo-reference electrodes drawn by 4B and 6B pencils were checked as they showed similar calibration curves in the case of Cu^2+^ determination. By increasing the carbon content of the pencil, a more uniform electrode coverage on the paper was seen (6B vs. 4B). For further studies, the 6B pencil was used in the pseudo-reference electrode drawing.

### 3.3. Inorganic Ions Determination

Determination of four inorganic ions including Cu^2+^, Cr^3+^, Ag^+^, and Hg^2+^ were done by carbon paste indicator electrodes without any modifier while in the case of Cd^2+^ measurement, benzo15-crown-5 (B15C5) was added to the paste.

The experimental conditions of the sensors were the same as reported in the original papers for conventional electrodes, so it would be possible to make a better comparison between ePAD and the previous reported sensors. Measuring the pH value of paper, which is explained in Supplementary Materials, showed that paper was almost neutral (≈6.8). Hence, pH does not play a significant role in measurement and the measurement conditions are similar to those of the bulk system [27,32,33,34,35].

#### 3.3.1. Unmodified Carbon Paste Electrode for Measurement of Cu^2+^, Ag^+^, Cr^3+^ and Hg^2+^ Ions

Ionic strength and pH were adjusted by capillary movement of 50.0 μL of 0.1 mol·L^−1^ KNO_3_ in 0.1 mol·L^−1^ HAc-NaAc buffer with a pH value of 5.0 in the sample channel. After dryness of the sensor at room temperature for 4.0 min, the sensor was folded so it was ready for ion determination. The bottom side of the sample channel was placed in the analyte solution so the sample would rise by the capillary effect to the top of the channel. By attachment of potentiometer alligators to the sensor electrodes, potential difference related to the ion concentration was read.

**Figure 2:**
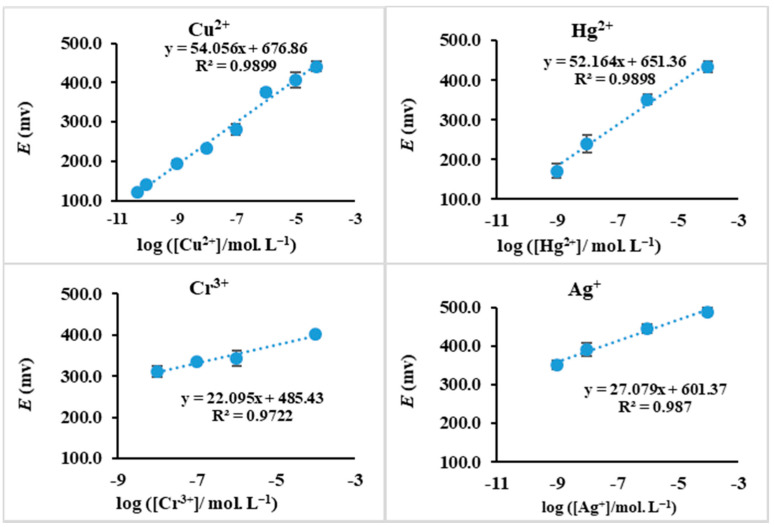
Calibration curves for Cu^2+^, Hg^2+^, Cr^3+^ and Ag^+^ ions with UCPE. Experimental conditions: 0.1 mol·L^−1^ KNO_3_ in 0.1 mol·L^−1^ HAc-NaAc buffer pH 5.0, room temperature, 6B pencil as a pseudo-reference electrode and CPE (72 wt % of graphite powder and 28 wt % of Nujol oil) as an indicator electrode.

The effective parameters (type and concentration of buffer, pH value, electrolyte, and carbon paste mixture) were all adjusted the same as the previously reported method for determination of Cu^2+^ ion by carbon paste ISE [32]. For more certainty, a general comparison between other options of electrolyte and buffer was performed in the case of Cu^2+^ determination by ePAD. As it can be seen in the Supplementary Materials, results indicate that the optimum condition for ePAD matches the reported bulk analysis conditions [32] (0.1 mol·L^−1^ acetate buffer pH 5.0 containing 0.1 mol·L^−1^ KNO_3_). This optimum condition was used for all studied metal ions in this section. The obtained calibration curves are given in Figure 2.

The results obtained from the experiments indicate a super-Nernstian response with slope of 54.1 and 52.2 mV·decade^−1^ for Cu^2+^ and Hg^2+^ respectively. A Nernstian behavior with a slope of 22.1 mV·decade^−1^ for Cr^3+^ and a sub-Nernstian response (slope 27.1 mV·decade^−1^) for Ag^+^ is seen. The slopes of Cu^2+^, Hg^2+^ and Cr^3+^are almost the same as those reported for these metal ions in the bulk solution analysis [27,32,33,34]. However, unlike previous reports, a sub-Nernstain behavior was observed for Ag^+^ determination. This can be attributed to the adsorption of Ag^+^ ions on the paper surface (Figure S8) [27]. So, the concentration of silver ion at the end of sampling channel, where the solution is in contact with electrodes, might be lower than that in the sample solution. In the other words, the electrode senses lower concentrations of Ag^+^ than in the bulk solution. Fortunately, as shown in Figure 2, this a reproducible behavior, an in the presence of adsorption, a well-defined calibration curve was obtained for Ag^+^.

**Figure 3:**
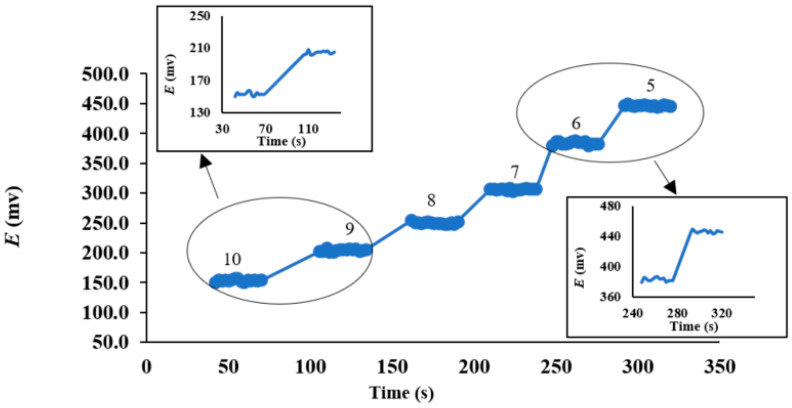
Dynamic response time of copper sensor at different concentration of Cu^2+^: The numbers denote the concentration (mol·L^−1^) of Cu^2+^ in pM (−log [Cu^2+^]) format. Experimental conditions: KNO_3_ 0.1 mol·L^−1^ in HAc-NaAc buffer 0.1 mol·L^−1^ pH 5.0, room temperature, 6B pencil as a reference electrode and CPE (72 wt % of graphite powder and 28 wt % of Nujol oil) as an indicator electrode. For each concentration, a separate sensor was used.

In Figure 3 are shown the response’s dynamic of the sensor at different concentrations of Cu^2+^. Beginning of potential reading was just after putting the end of sampling pad into sample solution. As seen the response time depends on the concentration of Cu^2+^ such that the response time was decreased by increasing in concentration. This plot shows that changes in the response of the sensor (even for trace concentrations of Cu^2+^) are significant comparing to the blank solution.

Comparison between the results of the reported bulk analysis [27] with the results for Cu^2+^ using ePAD, suggests that a wider linear range is accessible utilizing the origami paper-based ISE sensor (5.0 × 10^−11^–5.0 × 10^−5^ mol·L^−1^ by ePAD vs. 1.0 × 10^−6^–1.0 × 10^−3^ mol·L^−1^ in bulk analysis). Moreover, DL for Cu^2+^ is 4.0 × 10^−11^ by ePAD (Figure S9).

#### 3.3.2. Benzo15-Crown-5 Modified Carbon Paste Electrode for Measurement of Cd^2+^ Ion

The ability of crown ethers to complex with cations is well known [36]. The cavity size of the B15C5 (1.10 Å) suits well for the uptake of Cd^2+^ (radius 0.97 Å). Therefore, this crown ether had been used previously as a modifier in carbon paste electrode for sensing of Cd^2+^ [35].

For Cd^2+^ sensing by the ePAD, the indicator electrode was drawn using Benzo15-crown-5 modified carbon paste (B15C5-MCPE). For preparation of B15C5-MCPE, the same composition as the original work was used [35]. B15C5-MCPE includes 71% graphite, 25% Nujol oil and 4% B15C5. The hydrophilic area of pseudo-reference electrode was filled by 6B pencil. Ionic strength and the pH were adjusted by capillary movement of 50.0 μL of 0.1 mol·L^−1^ KNO_3_ in 0.1 mol·L^−1^ HAc-NaAc buffer with a pH value of 5.0 in the sample channel. After the dryness and folding of the sensor, the bottom side of sample channel was placed in the analyte solution.

**Figure 4:**
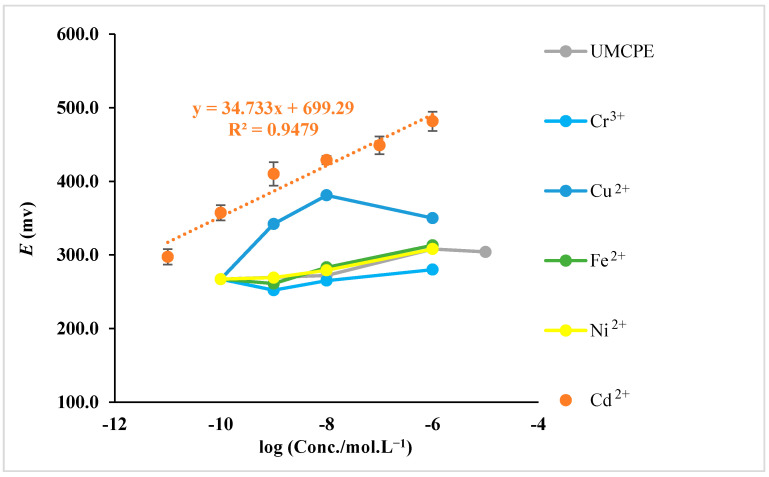
Investigation of B15C5-MCPE selectivity toward Cd^2+^ ion and Calibration curve for Cd^2+^ ion determination. Experimental conditions: channel containing 0.1 mol·L^−1^ KNO_3_ in 0.1 mol·L^−1^ HAcNaAc buffer pH 5.0; 6B pencil as a pseudo-reference electrode and B15C5-MCPE as an indicator electrode.

First, the response of the ePAD to different transition metal ions was investigated by obtaining the calibration curves (Figure 4). The potentiometric selectivity coefficient of the cadmium sensor was also determined by the separate solution method (SSM) (Table S3). It was observed that among the studied metal ions, the ePAD respond selectively toward Cd^2+^ ions. The obtained calibration curve for Cd^2+^ is shown in Figure 4, suggesting linearity in the concentration range from 1.0 × 10^−11^–1.0 × 10^−6^ mol·L^−1^. The slope of the calibration curve is 34.7 mV decade^−1^, showing a Nernstian behavior. The response time of this sensor was measured to be around 50 s, which is comparable with that of the bulk system (which was around 30 s).

Overall, this ePAD represented much higher sensitivity (DL of 6.3 × 10^−12^) compared to the previously conventional potentiometric cell (DL of 3.2 × 10^−5^) [35].

### 3.4. Bio-Sensing by ePAD

In this section, the usability of the ePAD in H_2_O_2_ and glucose determination is investigated and discussed.

#### 3.4.1. Measurement of H_2_O_2_ with MnO_2_ Modified Carbon Paste Electrode (MnO_2_-MCPE)

The main steps for measurement of H_2_O_2_ concentration were the same as those explained for inorganic ions determinations. However, the modifier in the indicator electrode was changed to the manganese dioxide and the pH adjustment was performed by 0.1 mol·L^−1^ NH_3_-NH_4_Cl buffer solution with pH 8.5 [37]. MnO_2_-MCPE prepared as same as previously reported [37], which included 71% graphite, 25% Nujol oil and 4% MnO_2_. The SEM image of MnO_2_-MCPE is shown in Figure S10B. A mixture of needle-shaped and flake-shaped of MnO_2_ with extended surface area is obvious compared to the unmodified carbon paste electrode.

**Figure 5:**
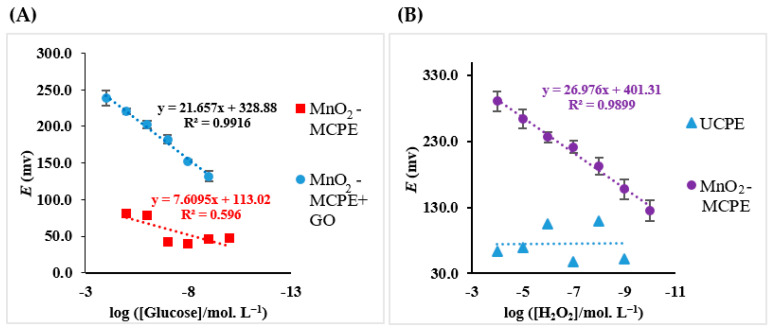
(**A**) Calibration curves for H_2_O_2_ using different indicator electrode composition; UCPE (blue triangles) and modified CPE (71 wt % of graphite powder, 25 wt % of Nujol oil and 4 wt % of manganese dioxide) (purple circles). (**B**) Calibration curve for glucos represented in the presence (blue circle) and absence (red square) of GOx. Experimental conditions: NH_3_-NH_4_Cl buffer solution 0.10 mol·L^−1^ with pH = 8.5, 6B pencil as a pseudo-reference electrode and modified CPE (71 wt % of graphite powder, 25 wt % of Nujol oil and 4 wt % of manganese dioxide) as an indicator electrode.

The potentiometric response of the ePAD to the H_2_O_2_ standard solutions of different concentration was evaluated. The calibration curve was obtained by plotting potential responses against logarithm of H_2_O_2_ concentration. As can be seen from Figure 5A, a linear regression with the slope of 26.98 mV decade^−1^ exists in the H_2_O_2_ concentration range of 1.0 × 10^−4^–1.0 × 10^−10^ mol·L^−1^. The unmodified CPE was not sensitive to the changes in H_2_O_2_ concentration. The analytical appraisals of ePAD are compared with those of bulk potentiometry in Table S4. Similar to the metal ion sensing, much higher sensitivity has been achieved by ePAD with DL of 4.0 × 10^−11^ mol·L^−1^. The response time of ePAD for H_2_O_2_ sensing is 12 s, which is comparable to bulk potentiometry.

The possible response mechanism of MnO_2_-MCPE toward H_2_O_2_ has been explored previously by Zhen and Gao [37]. Briefly, H_2_O_2_ oxidize MnO_2_ to produce MnO_4_^2−^ then the organic materials existed in the paraffin oil reduces the MnO_4_^2−^ to MnO_2_. As a result, the potentiometric response of this sensor is probably a mixture of redox potential between the MnO_4_^2−^ and MnO_2_.

#### 3.4.2. Using ePAD As a Biosensor for Glucose

In order to prepare a biosensor for glucose, glucose oxidase enzyme (GOx) was fixed on the MnO_2_-MCPE indicator electrode. To do so, a 2.5 µL of GOx solution, which was prepared in 0.1 mol·L^−1^ phosphate buffer pH = 7.4, was dropped caste on the surface of the indicator electrode. The enzyme concentration was optimized by fixing GOx solutions with various concentrations on the electrode and observing the responses of the sensors to the glucose sample with 1.0 µmol L^−1^ concentration. Four various GOx concentrations of 0.24, 0.74, 1.24 and 1.72 U µL^−1^ were tested. The results (Figure S11) showed an improvement in the response by increasing the Gox concentration up to 1.24 U µL^−1^. Further additions in GOx concentration showed no effect on the recorded potential response. As a result, 1.24 U µL^−1^ had been chosen as an optimum GOx concentration.

The changes in potential difference of ePAD as a function of glucose concentration were monitored. For comparison, the response of ePADs without the addition of GOx was also measured. The results are given in Figure 5B, representing a well-defined linear relationship for glucose concentration in the range of 1.0 × 10^−9^–1.0 × 10^−4^ mol·L^−1^. However, in the absence of GOx, the ePAD did not respond to the glucose variations. Similar to the other studied analytes, the ePAD represented a super sensitivity to glucose with DL of 4.6 × 10^−10^. The response time was 15–25 s, which is comparable to the previously reported glucose biosensor [38,39,40].

To reduce sample consumption in the glucose determination, the sensor dimension was decreased to the one-third of the original size (Figure S12). As a result, the device preserved its performance while the sample volume needed for the analysis was reduced from 50.0 to 10.0 μL and the response time was decreased e.g., from 20 s to 15 s for 1.0 × 10^−5^ mol·L^−1^ of glucose.

The sensitivity of the sensor (slope in calibration curves) during this interval, was chosen as a factor representing the sensor stability. As it can be seen from Table S6, the sensor is stable for 17 days after preparation. In longer times, the sensor sensitivity is reduced and also the sensor needs more time to provide the consistent potential difference as a sensor response.

#### 3.4.3. Real Sample Analysis

Recently, attentions have been directed toward the development of non-invasive methods for diabetes monitoring. For non-invasive measurements, the glucose level in body fluids other than blood (such as urine, tear, sweat, and saliva) should be measured. However, the glucose level in these fluids is very low (<8.0 × 10^−4^ mol·L^−1^) [41] and most of the developing biosensors for glucose suffer from low sensitivity. The super-sensitivity of our ePAD allows non-invasive measurement of glucose in biological fluids other than blood. To do so, glucose testing was performed on various body fluid samples such as a tear, blood, and urine.

The results of glucose concentration values in Table 1 showed an excellent agreement with the reference method (Bionime GM110 Blood glucose monitor).

A commercial Tearlose tear solution and artificial human urine were prepared [41]. The procedure for preparation of artificial urine is explained in the Supplementary Materials. The defined concentration of glucose was spiked to these solutions and then the recovery values were obtained by comparing the spiked and the sensor determined amounts. Recoveries from 86.0% to 110.0% expresses appropriate performance of the designed ePAD (Table 2).

### 3.5. Comparison with Other Microfluidics Devices

A comparison between our ePAD and some of recently published paper-based microfluidic glucose sensors is given in Table 3. It can be observed that the detection limit and response time of our device has been improved compared to other paper-based sensors. Moreover, sample volume used by this ePAD, has been reduced compared to most of the assays, which is a great advantage in biological studies.

## 4. Conclusions

A 3D origami paper-based potentiometric device had been introduced. The origami design of the sensor made it possible to have lower limits of detection for inorganic ions and also biological analytes. The selectivity of the sensor response to some analytes was improved by the addition of modifiers to the indicator electrode. A glucose biosensor with super sensitivity was obtained by using MnO_2_–modified carbon paste electrode, which measured H_2_O_2_, and addition of glucose oxidase, which convert glucose to H_2_O_2_. This ultra-sensitivity allowed measurement of glucose in biological fluids other than blood (urine and tear). In addition, reproducibility and response time of the sensor is improved compared to the bulk analysis. The accurate results obtained for measurement of a very low level of glucose in these fluids, suggests that the ePAD biosensor has the potential to be used for non-invasive monitoring of diabetes.

## Figures and Tables

**Table 1:** Concentration of glucose predicted by ePAD biosensor and commercial blood glucometer.

**Commercial Glucometer (mmol·L^−1^)**	**ePAD Biosensor (mmol·L^−1^)**	**%Relative Error**
7.5	6.7	−10.7
5.8	5.4	−6.9
7.4	7.5	+1.4
5.0	4.8	−4.0

**Table 2:** Result of recovery studied of glucose in tear solution and an artificial human urine.

Sample	Added (μmol·L^−1^)	Found (μmol·L^−1^)	%Recovery	%Relative Error
Artificial urine	0.0010	0.0011	110	10.0
	1.00	1.07	107	7.0
	10.00	8.64	86.4	−13.6
Artificial tear	0.0010	0.00098	97.7	−2.0
	0.010	0.0099	98.9	−1.0
	10.00	11.20	112	12

**Table 3:** A comparison between this work and several electrochemical ePADs for measurement of glucose.

Sensors	Linear Range (mol·L^−1^)	DL (mol·L^−1^)	Response Time (s)	Ref.
Nafion/GOx/platinized filter paper	1.0 × 10^−4^–3.2 × 10^−3^	3.2 × 10^−5^	50	[42]
platinized filter paper/Nafion/GOx	3 × 10^−4^–3 × 10^−3^	1 × 10^−4^	60	[37]
platinised paper/polyvinyl alcohol/chitosan/GOx	3.0 × 10^−5^ to 1.0 × 10^−3^	2.0 × 10^−5^	20–30	[39]
Au dendrites/Whatman filter papers	1.0 × 10^−5^–1.5 × 10^−2^	6 × 10^−7^	240	[43]
MnO_2_-doped CPE/GOx	1.0 × 10^−9^–1.0 × 10^−4^	4.6 × 10^−10^	15–25	In this work

## Data Availability

Not applicable.

## Supplementary Materials

The following are available online at https://www.mdpi.com/2079-6374/11/2/44/s1, Figure S1: Photograph of a potentiometric paper-based ion selective sensor. The reference and indicator electrodes were positioned vertical and horizontal, respectively. (The reference electrode is located behind the paper and under clothespins); Figure S2. Pictures of the sensors with different width of sample channel: (A) Without sample channel; (B) The sample channel which is wider than electrode width; (C) Channel width equal to the electrode’s and (D) The sample channel thinner than the electrode width; Figure S3. Different volumes of murexide solution was loaded and then R values (in RGB space) of the first, end and the middle sections of the channel; Figure S4. Image of sensor with (a) 20.0 μL (b) 30.0 μL and (c) 50.0 μL of murexide solution; Figure S5. Pictures of sensors in different designs A, B, C the number of layers is odd and D, E, F, G the number of layers is even; Figure S6. Effect of the type of pencil used as reference electrode on the performance of the potentiometric ePAD: (A), (B), (C), (D), (E) and (F) related to 6B, 4B, 3B, 9B, HB and 4H pencil leads, respectively. Experimental conditions: KNO_3_ 0.1 mol·L^−1^ in HAc-NaAc buffer 0.1 mol L^−1^ pH 5.0, room temperature, 6B pencil as a reference electrode and CPE (72 wt % of graphite powder and 28 wt % of Nujol oil) as an indicator electrode; Figure S7. SEM images of surface of electrode. Reference electrode with (A) 6B and (B) 4B pencil and indicator electrode with UCPE; Figure S8. Possible conformation of the compound formed from cellulose binding to silver; Figure S9. Detection limit for Cu^2+^ ion. Experimental conditions: KNO_3_ 0.1 mol·L^−1^ in HAc-NaAc buffer 0.1 mol·L^−1^ pH 5.0, room temperature, 6B pencil as a reference electrode and CPE (72 wt % of graphite powder and 28 wt % of Nujol oil) as an indicator electrode; Figure S10. The SEM image of surface of working electrode with A) UCPE and B) MnO_2_ modified CPE; Figure S11. Optimization of GOx concentration in phosphate buffer (0.1 mol·L^−1^) by fixing glucose concentration at 1.0 µM. Experimental conditions: NH_3_-NH_4_Cl buffer solution 0.10 mol·L^−1^ with pH = 8.5, 6B pencil as a reference electrode and modified CPE (72 wt % of graphite powder, 25 wt % of Nujol oil and 4 wt % of manganese dioxide) as an indicator electrode; Figure S11. The image of sensor in different sizes; Figure S13. Comparison between different conditions of electrolyte and identical buffer in Cu^2+^ determination by ePAD. (A) NaCl 1.0 mol L^−1^ in HAc-NaAc buffer 0.1 mol L^−1^ (B) KNO_3_ 0.01 mol L−1 in HAc-NaAc buffer 0.1 mol L^−1^ (C) KNO_3_ 0.1 mol L−1 in HAc-NaAc buffer 0.1 mol L^−1^. 6B pencil as a reference electrode and CPE (72 wt % of graphite powder and 28 wt % of Nujol oil) as an indicator electrode. Table S1. Effect of the size of sample channel width on the precision of potentiometric measurements (1.0 × 10^−6^ mol·L^−1^ of Cu^2+^ and three times repeat for each design). Experimental conditions: KNO_3_ 0.1 mol·L^−1^ in HAc-NaAc buffer 0.1 mol·L^−1^ pH 5.0 and room temperature; Table S2. Analytical characterization of the sensor with different junction layer design. Experimental conditions: KNO_3_ 0.1 mol·L^−1^ in HAc-NaAc buffer 0.1 mol·L^−1^ pH 5.0, room temperature and difference concentration of copper (1.0 × 10^−5^–1.0 × 10^−9^ mol·L^−1^); Table S3. Potentiometric selectivity coefficient of the Cd^2+^ ion using SSM. Experimental conditions: KNO_3_ 0.1 mol·L^−1^ in HAc-NaAc buffer 0.1 mol·L^−1^ pH 5.0, room temperature. 6B pencil as a reference electrode and modified CPE (mixing 71 wt % of graphite powder, 25 wt % of Nujol oil and 4% B15C5) as an indicator electrode; Table S4. A comparison between the ePAD in this work and various bulk potentiometric method for measurment of H_2_O_2_; Table S5. The effect of the hydrophobicity of the back of the working electrode on the precision of potentiometric measurements (1.0 × 10^−6^ mol·L^−1^ of Cu^2+^ and repeated three times for each design). Experimental conditions: KNO_3_ 0.1 mol·L^−1^ in HAc-NaAc buffer 0.1 mol·L^−1^ pH 5.0 and room temperature; Table S6. Stability of glucose sensor (The response of sensor was recorded in the presence of three concentration of glucose). Experimental conditions: NH_3_-NH_4_Cl buffer solution 0.10 mol L^−1^ with pH = 8.5. Sensor was kept in the dark condition before use.
