# Supplementary material for: An All-in-One Solid State Thin-Layer Potentiometric Sensor and Biosensor Based on Three-Dimensional Origami Paper Microfluidics
Source: Biosensors (Basel). 2021 Feb 10;11(2):44. doi: 10.3390/bios11020044 (PMC7916752; doi:10.3390/bios11020044)
Supplement: Supplementary file 1 [file biosensors-11-00044-s001.pdf]

Supporting information

An All-in-One Solid State Thin-Layer Potentiometric Sensor Based on Three-Dimensional Origami Paper Microfluidics for Ultrasensitive Detections

Shiva Pesaran, Elmira Rafatmah and Bahram Hemmateenejad*

Chemistry Department, Shiraz University, Shiraz, 71454, Iran

*:Corresponding Author:

E-mails: hemmatb@shirazu.ac.ir, hemmatb@sums.ac.ir.

Tel.: 0098-713 646 0724.

Fax: 0098-713 646 0788.

Materials and method

D-Fructose, sucrose, D-maltose, D-galactose, lactose, glycogen, graphite powder, MnO_2, $KMnO_4$, $Na_2C_2O_4$, NH_4Cl, $Cd(NO_3)_2.4H_2O$, K_2HPO_4, Nujol oil, benzo15-crown-5, $Fe(NO_3)_3{\cdot}9H_2O$, $Ni(NO_3)_2.6H_2O$, KNO_3, sodium acetate, urea, uric acid, NaCl, KCl, $CaCl_2.2H_2O$, $MgSO_4.7H_2O$, $NaHCO_3$, Na_2SO_4,Na_2HPO_4, $NaH_2PO_4.H_2O$, ammonia solution 25%, hydrogen peroxide 30% and murexide were all purchased from Merck. KH_2PO_4, creatinine, $Cu(NO_3)_2{\cdot}3H_2O$, $AgNO_3$, $Hg(NO_3)_2.H_2O$, $[Cr(H_2O)_6]NO_3)_3.3H_2O$ were purchased from Fluka. L(+)-Cysteine and Glucose oxidase (GOx), from Aspergillus niger type II, were purchased from Riedel-de-Haën and Sigma-Aldrich respectively.

$Na_3C_6H_5O_7.2H_2O$, D-glucose and glacial acetic acid were purchased from BDH Chemicals while Schleicher & Schuell® (s&s) Grade 2040b qualitative filter paper (with a thickness of is 0.2 mm) was used for device fabrication. Carbon nanotube (CNT) was a gift of Dr. Doroodmand's lab. Koh-i-Noor Hardtmuth pencils 3B, 4B, 6B, 9B, 4H and HB of different commercial brands were collected from local stores.

All solutions were prepared using double deionized water.

The artificial urine was prepared by mixing 2.427 g of urea, 0.034 g of uric acid, 0.090 g of creatinine, 0.297 g of $Na_3C_6H_5O_7$_$2H_2O$, 0.634 g of NaCl, 0.450 g of KCl, 0.161 g of NH_4Cl, 0.089 g of $CaCl_2.2H_2O$, 0.100 g of $MgSO_4.7H_2O$, 0.034 g of $NaHCO_3$, 0.003 g of NaC_2O_4, 0.258 g of Na_2SO_4, 0.100 g of $NaH_2PO_4.H_2O$, and 0.011 g of Na_2HPO_4 in 200.0 mL of deionized water [1].

Apparatus

A HP LaserJet 1320 printer from HP was used to print the devices. A Memmert Incubator Oven INB200 was used for curing the printed μPADs. Electrochemical measurements were made using an AZ-86502 bench top pH meter at room temperature (25.0 °C), Bionime GM110 Blood glucose monitor was used as reference method.

A lab-made potentiometer was used for wifi sending of potential data on a mobile phone.

The scanning electron micrographs (SEM) were obtained with a TESCAN model VEGA3 instrument.

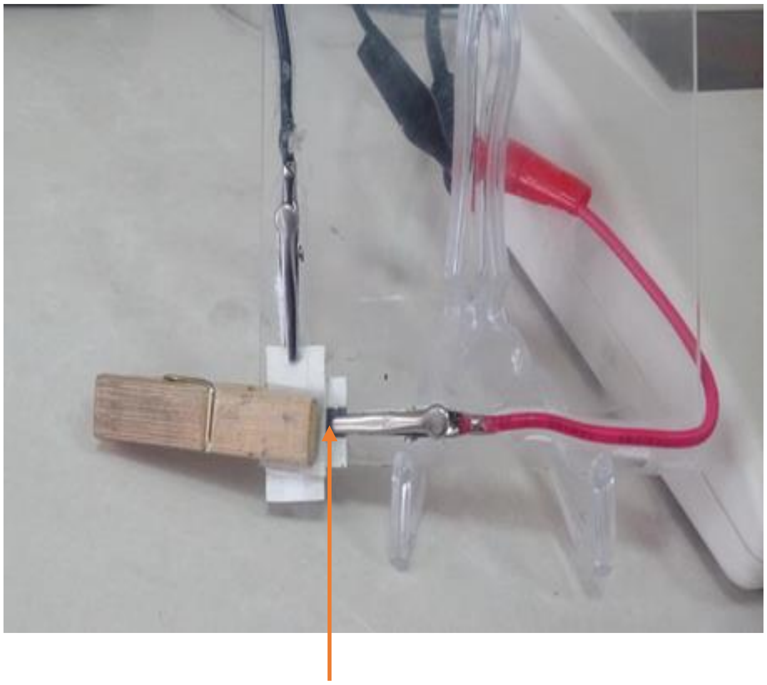

Figure S1. Photograph of a potentiometric paper-based ion selective sensor. The reference and indicator electrodes were positioned vertical and horizontal, respectively. (The reference electrode is located behind the paper and under clothespins)

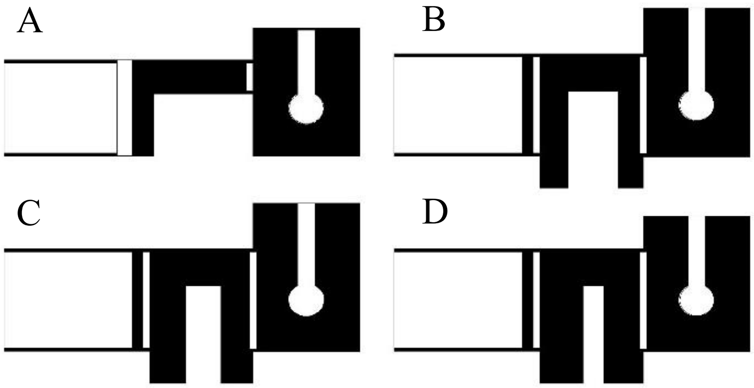

Figure S2. Pictures of the sensors with different width of sample channel: A) Without sample channel; B) The sample channel which is wider than electrode width; C) Channel width equal to the electrode's and D) The sample channel thinner than the electrode width.

Table S1. Effect of the size of sample channel width on the precision of potentiometric measurements (1.0×10^{-6} mol.L^{-1} of Cu^{2+} and three times repeat for each design). Experimental conditions: KNO_3 0.1 mol.L^{-1} in HAc-NaAc buffer 0.1 mol.L^{-1} pH 5.0 and room temperature.

**Sample channel size relative to electrode width**	**Potential**	**Potential**	**Potential**	**RSD%**
Same size	326.0	335.0	345.0	2.8
Thicker	314.0	350.0	265.0	13.8
Thinner	Variable	Variable	Variable	-
No sample channel	237.0	172.0	137.0	23.4

Sample volume

The sample volume which was loaded to the sample channel was set to be as much as the channel becomes saturated from the sample. The needed volume for this purpose was measured by a colorimetric analysis. Therefore, chromatic substance like murexide was selected to evolution of the volume of the sample channel. Different volumes of murexide solution was loaded into sample channel and then color intensity (R in RGB space) at three parts of channel (start, end and middle sections of the channel) was measured. The RSD% values of these three R values were calculated. As it is obvious from Figure S3, by increasing the sample volume, the concentration gradient of murexide and therefore RSD% in the channel would be decreased. The final sample volume of 50.0 µL was chosen for further optimizations. (Figure S4 show that sensors with different volume of murexide solution).

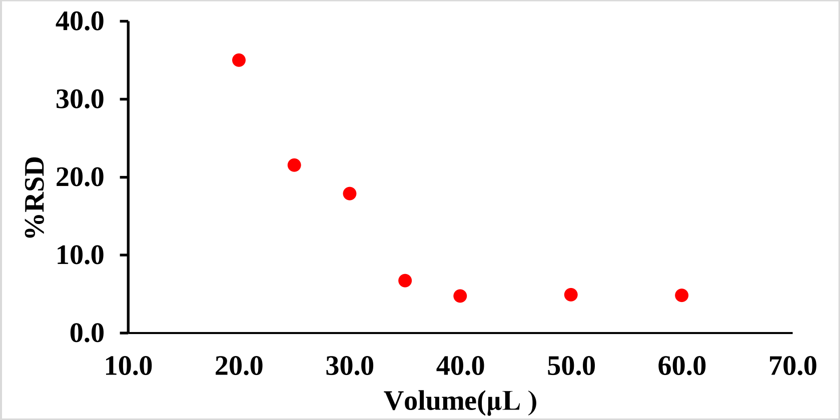

Figure S3. Different volumes of murexide solution was loaded and then R values (in RGB space) of the first, end, and the middle sections of the channel.

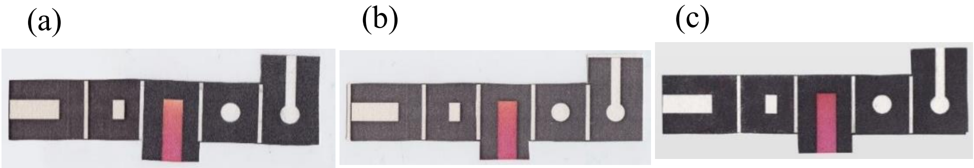

Figure S4. Image of sensor with a) 20.0 µL b) 30.0 µL and c) 50.0 µL of murexide solution

The number of junction layers were examined to see if it had any effect on the results. Seven different designs were investigated (Figure S5) (A) both layers of square and circular hydrophilic parts were duplicated; (B) sensor contains one layer from each of square and circular hydrophilic parts; (C) elimination of these two layers from the sensor; (D, E) sensor containing just one of these layers (circular and square hydrophilic parts respectively); (F, G) sensor with two same circular or square junction layers. For each design a calibration curve was developed for Cu^{2+} ion in the concentration ranges of $1.0 \times 10^{-5} – 1.0 \times 10^{-9}$ mol.L^{-1}. Comparison of the calibration curves (Table S2) showed that the best results were obtained when design "B" was employed. In order to choose the optimal design, it should be noted that if the number of layers is even and the paper is folded in the form of an origami, the working electrode (or reference) is facing outside and it is attached to clothespins. From among designs A, B and C, design B was selected. Design C has a less correlation coefficient than design B; also, when using volumes greater than 50.0 μL (to ensure uniformity of concentration across the channel), hydrophobic parts around the electrode lose their hydrophobic properties. Design A not only has a longer response time than design but also more volume of consumable solution.

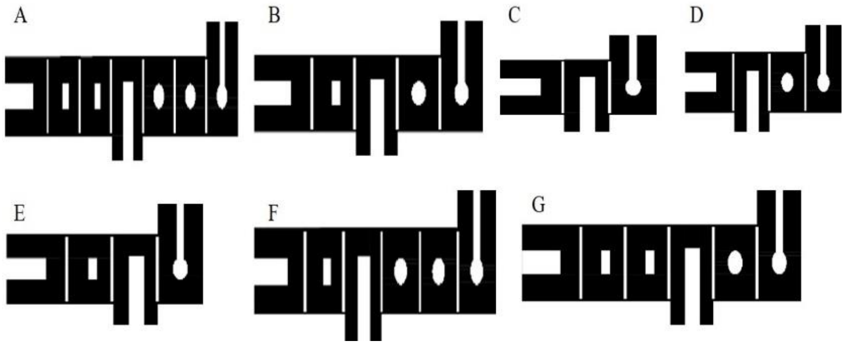

Figure S5. Pictures of sensors in different designs A, B, C the number of layers is odd and D, E, F, G the number of layers is even

Table S2. Analytical characterization of the sensor with different junction layer design. Experimental conditions: KNO_3 0.1 mol.L^{-1} in HAc-NaAc buffer 0.1 mol.L^{-1} pH 5.0, room temperature and difference concentration of copper (1.0×10^{-5} – 1.0×10^{-9} mol.L^{-1}).

Sensor	Number of junction layers	Sensitivity (mV·decade^{-1})	R^2
A	7	37.6	0.96
B	5	56.9	0.97
C	3	51.7	0.90
D	4	29.7	0.96
E	4	37.7	0.96
F	6	7.2	0.90
G	6	17.3	0.73

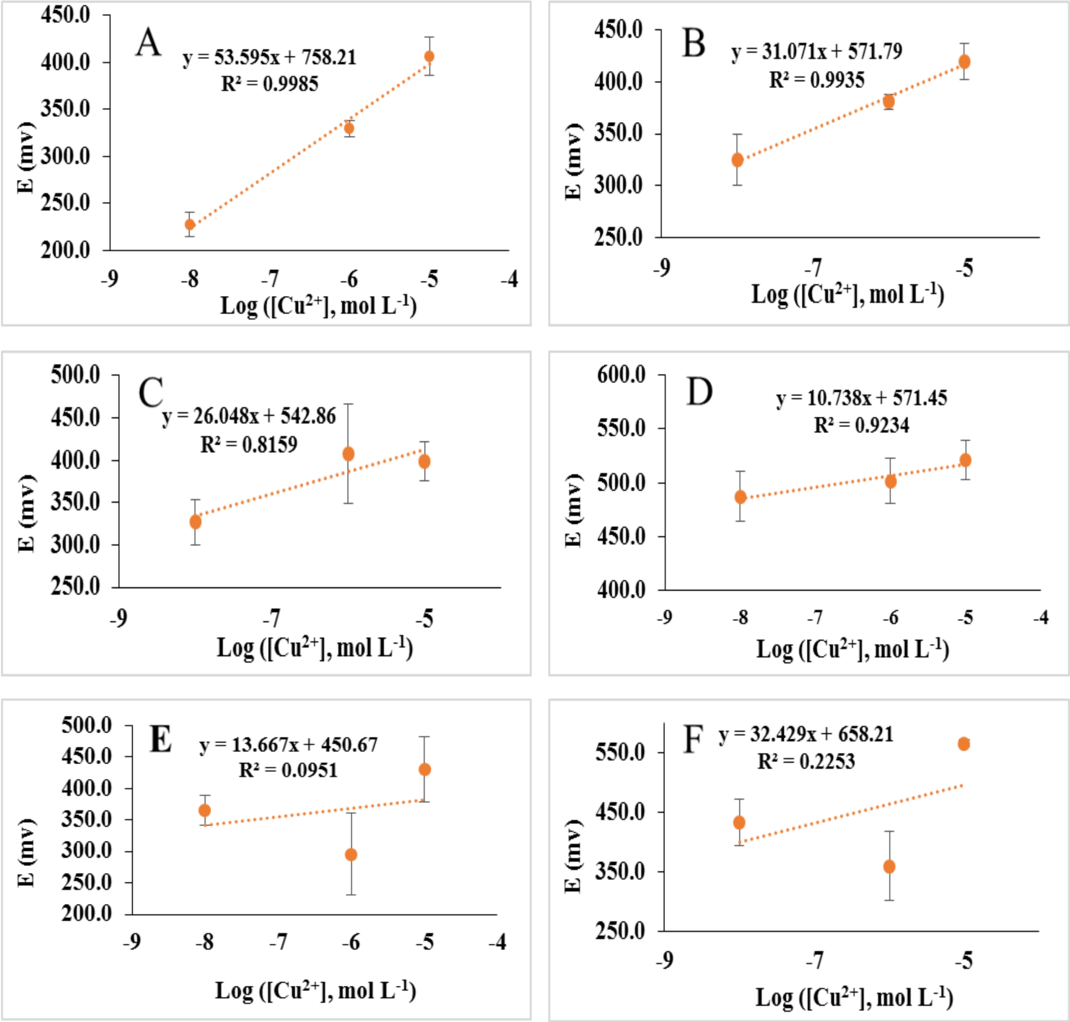

Figure S6. Effect of the type of pencil used as reference electrode on the performance of the potentiometric ePAD: (A), (B), (C), (D), (E) and (F) related to 6B, 4B, 3B, 9B, HB and 4H pencil leads, respectively. Experimental conditions: KNO_3 0.1 mol.L^{-1} in HAc-NaAc buffer 0.1 mol L^{-1} pH 5.0, room temperature, 6B pencil as a reference electrode and CPE (72 wt % of graphite powder and 28 wt % of Nujol oil) as an indicator electrode.

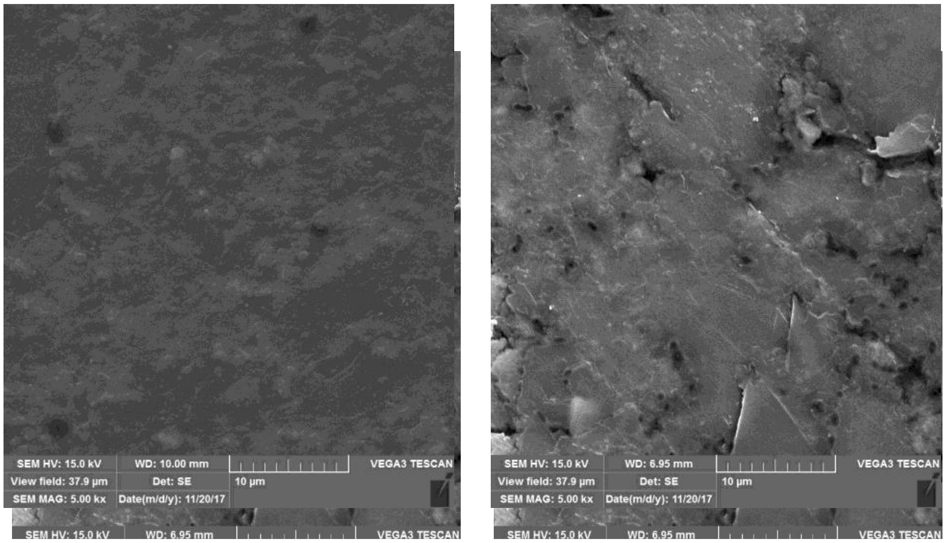

Figure S7. SEM images of surface of electrode. Reference electrode with A) 6B and B) 4B pencil and indicator electrode with UCPE.

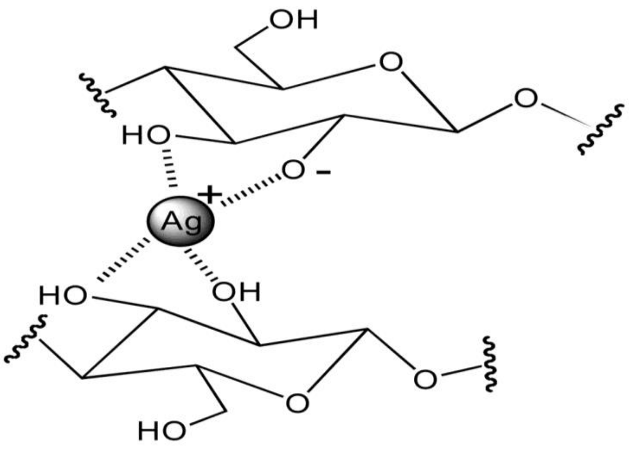

Figure S8. Possible conformation of the compound formed from cellulose binding to silver

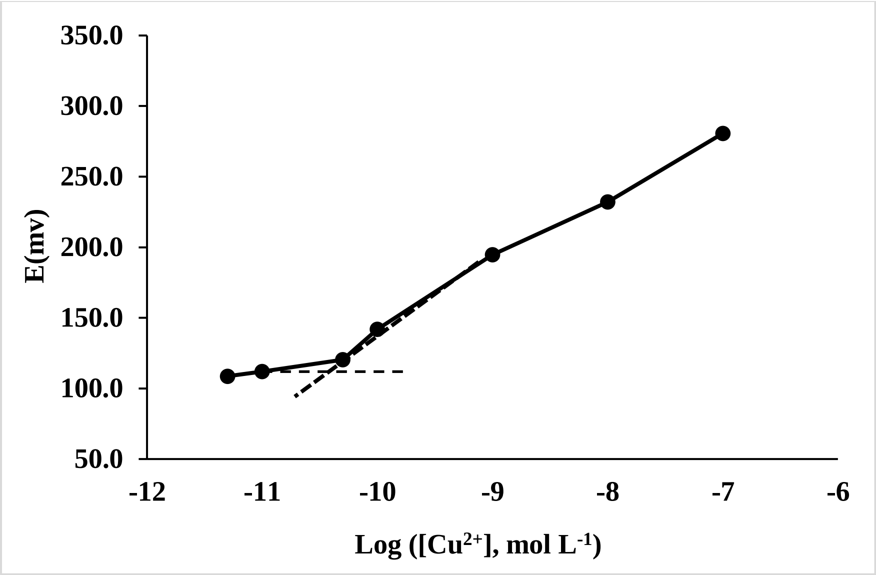

Figure S9. Detection limit for Cu^{2+} ion. Experimental conditions: KNO_3 0.1 mol.L^{-1} in HAc-NaAc buffer 0.1 mol.L^{-1} pH 5.0, room temperature, 6B pencil as a reference electrode and CPE (72 wt % of graphite powder and 28 wt % of Nujol oil) as an indicator electrode

Table S3. Potentiometric selectivity coefficient of the Cd^{2+} ion using SSM. Experimental conditions: KNO_3 0.1 mol.L^{-1} in HAc-NaAc buffer 0.1 mol.L^{-1} pH 5.0, room temperature. 6B pencil as a reference electrode and modified CPE (mixing 71 wt % of graphite powder, 25 wt % of Nujol oil and 4% B15C5) as an indicator electrode.

Interfering ion	$LogK_{A,B}$
Ni^{2+}	-5.9
Fe^{3+}	-1.7
Cr^{3+}	-8.7
Cu^{2+}	-8.4

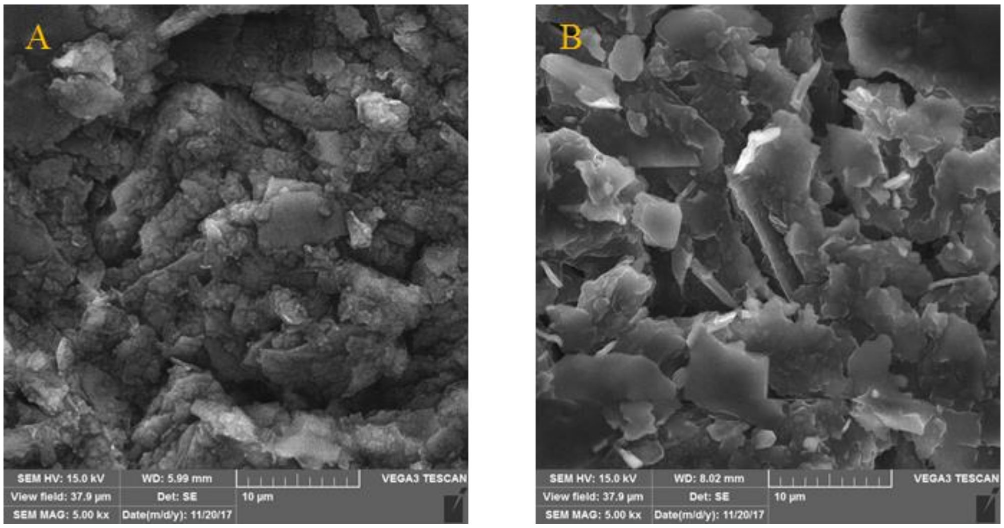

Figure S1010. The SEM image of surface of working electrode with A) UCPE and B) MnO_2 modified CPE.

Table S4. A comparison between the ePAD in this work and various bulk potentiometric method for measurment of H_2O_2.

**IEs**[1]	**Linear range (μmol.L^{-1})**	**DL (μmol.L^{-1})**	**Response time (s)**	**References**
MnO_2-doped CPE	0.3 -360.0	0.12	<180	[2]
GC[2]	2.1 -1100.0	-	<240	[3]
Gold- ORP[3]	-	0.40	30	[4]
VNN[4]	100000.0–0.1	25.00	-	[5]
FISE- HRP[5]	1.0 –200.0	0.80	10-40	[6]
calix[4]arene	550.0- 63000.0	40.00	240	[7]
MnO_2-doped CPE	100.0 -0.0001	0.000040	12-20	In this work

[1]IEs = Indicator electrodes
[2]GC = Glassy carbon
[3]Gold-ORP = gold-plated oxidation-reduction potential electrode
[4]VNN = 4-vinylphenylboronic acid - Nafion solution
[5]FISE- HRP = fluoride ion-selective electrode - horseradish peroxidase enzyme

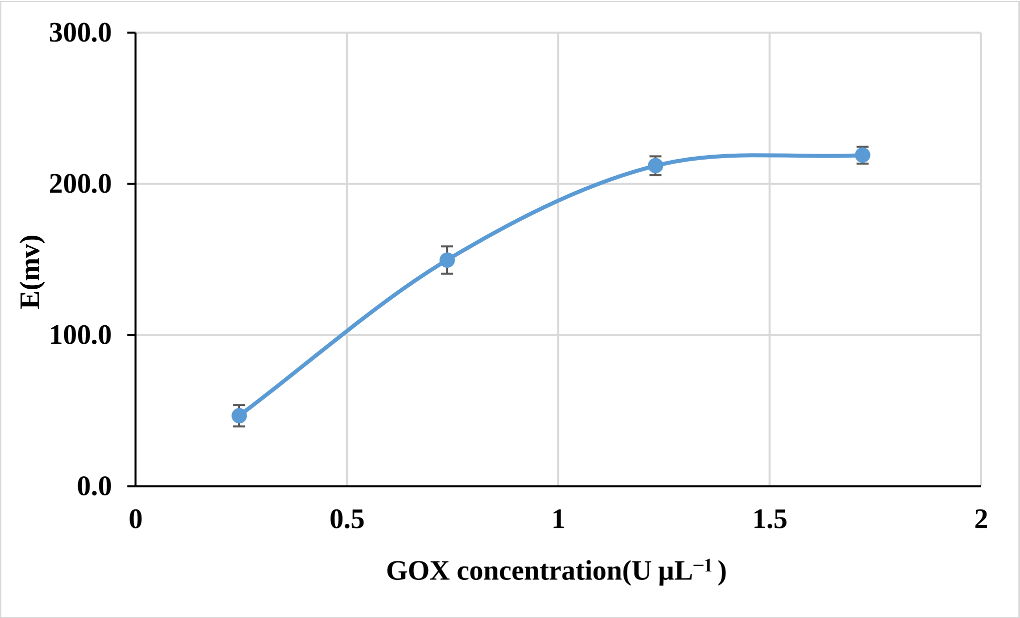

Figure S1111. Optimization of GOx concentration in phosphate buffer (0.1 mol.L^{-1}) by fixing glucose concentration at 1.0 µM. Experimental conditions: NH_3-NH_4Cl buffer solution 0.10 mol.L^{-1} with pH= 8.5, 6B pencil as a reference electrode and modified CPE (72 wt % of graphite powder, 25 wt % of Nujol oil and 4 wt % of manganese dioxide) as an indicator electrode.

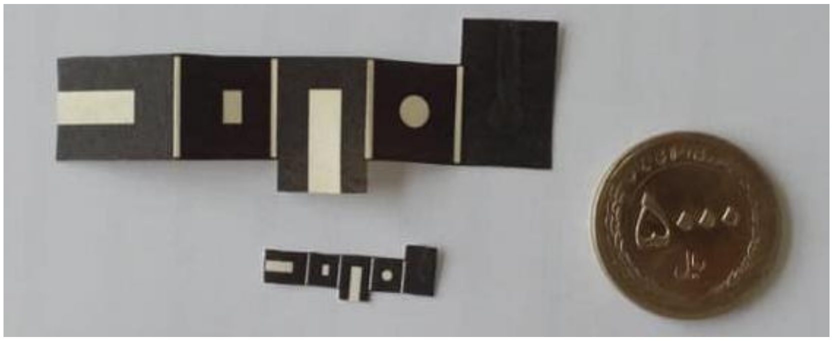

Figure S1212. The image of sensor in different sizes

3.3 Determination of the pH value of papers

The paper was cut into pieces of roughly 1.0 cm^2. The weighted, air-dry paper was transfer to a 100.0 mL beaker and then added 20.0 mL of distilled water and macerated with a flattened stirring rod until the specimen is uniformly wet. After that more 50.0 mL of the distilled water was added, stirred well, then covered with a watch glass, and allow to stand approximately 20.0 hour. The entire procedure is carried out at room temperature. After stirring the mixture once more, the pH of the unfiltered mixture was measured with the pH-meter equipped with a glass electrode [8].

After selecting the design of Figure S4B as the optimal design up to this step, the hydrophobicity of the back of the working electrode was evaluated. As shown in Table S5, back of the working electrode was printed to be hydrophobe. In addition, less volume of solution was consumed and RSD percentage decreased.

Table S5. The effect of the hydrophobicity of the back of the working electrode on the precision of potentiometric measurements (1.0×10^{-6} mol.L^{-1} of Cu^{2+} and repeated three times for each design). Experimental conditions: KNO_3 0.1 mol.L^{-1} in HAc-NaAc buffer 0.1 mol.L^{-1} pH 5.0 and room temperature

		**Potential**		**%RSD**
a	326.0	335.0	345.0	2.8
b	319.0	329.0	331.0	1.9

a: Back of the working electrode was not Hydrophobe
b: Back of the working electrode was Printed to be Hydrophobe

To be able to compare the ePAD results with the bulk analysis, the effective parameters (type and concentration of buffer, pH value, electrolyte and carbon paste mixture) were all adjusted the same as the previous reported method for determination of Cu^{2+} ion by carbon paste ISE [9]. For more certainty, a general comparison between 3 different conditions electrolyte and identical buffer in Cu^{2+} determination by ePAD were performed ((A) 0.1 mol.L^{-1} acetate buffer- 1.0 mol.L^{-1} NaCl [10], pH 5.0 &(B) 0.1 mol.L^{-1} acetate buffer- 0.01 mol.L^{-1} KNO_3, pH 5.0 & (C) 0.1 mol.L^{-1} acetate buffer- 0.1 mol.L^{-1} KNO_3, pH 5.0) (Figure S14). Results indicate that the optimum condition for ePAD matches the reported bulk analysis conditions [9] (0.1 mol.L^{-1} acetate buffer- 0.1 mol.L^{-1} KNO_3, pH 5.0).

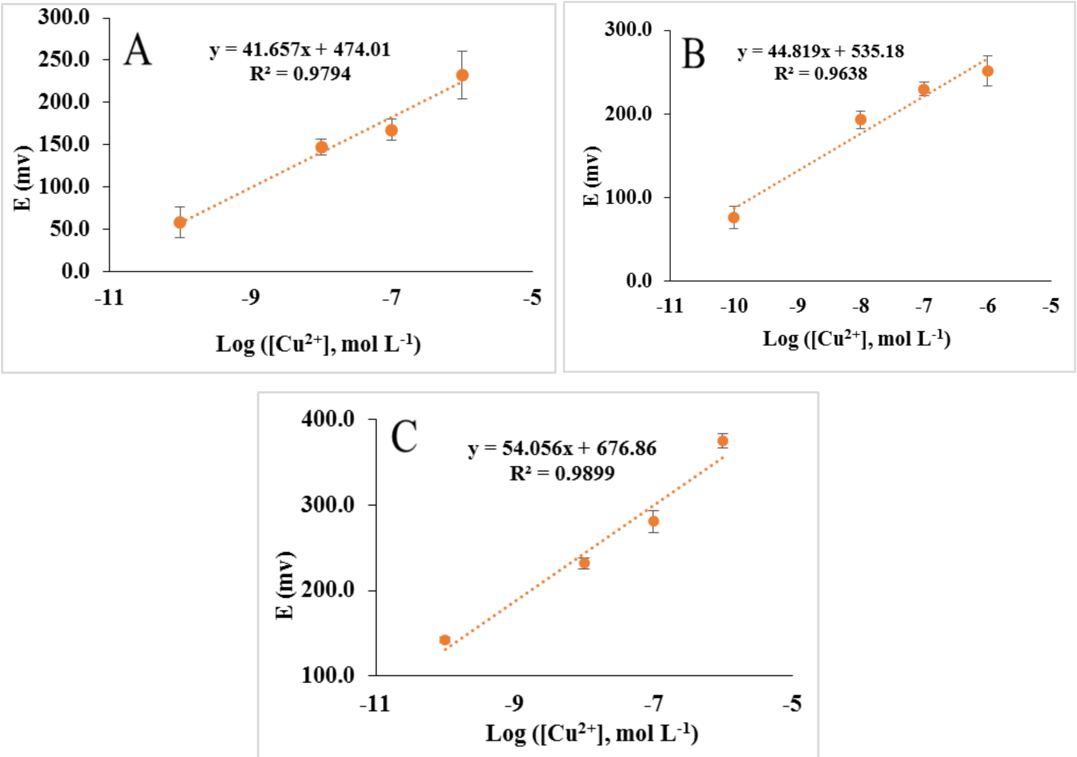

Figure S13. Comparison between different conditions of electrolyte and identical buffer in Cu^{2+} determination by ePAD. A) NaCl 1.0 mol L^{-1} in HAc-NaAc buffer 0.1 mol L^{-1} B) KNO_3 0.01 mol L^{-1} in HAc-NaAc buffer 0.1 mol L^{-1} C) KNO_3 0.1 mol L^{-1} in HAc-NaAc buffer 0.1 mol L^{-1}. 6B pencil as a reference electrode and CPE (72 wt % of graphite powder and 28 wt % of Nujol oil) as an indicator electrode.

Table S6. Stability of glucose sensor (The response of sensor was recorded in the presence of three concentration of glucose). Experimental conditions: NH_3-NH_4Cl buffer solution 0.10 mol L^{-1} with pH= 8.5. Sensor was kept in the dark condition before use.

DAY	Sensitivity (mV·decade^{-1})	Response time (s)
1	19	20-25
5	19.5	20-28
11	19	30-35
17	19.5	40-60
21	6.4	>120

References

1. Chutipongtanate S, Thongboonkerd V. Systematic comparisons of artificial urine formulas for in vitro cellular study. *Anal. Biochem.* **2010**;*402*:110-112.

2. Zheng X, Guo Z. Potentiometric determination of hydrogen peroxide at MnO_2-doped carbon paste electrode. *Talanta* **2000**;*50*:1157-1162.

3. Awad M.I, Oritani T, Ohsaka T. Simultaneous potentiometric determination of peracetic acid and hydrogen peroxide. *Anal. Chem.* **2003**;*75*:2688-2693.

4. Ohura H, Imato T, Yamasaki S, Ishibashi N. Potentiometric flow-injection determination of trace hydrogen peroxide based on its induced reaction in iron (III)-iron (II) potential buffer containing bromide and molybdenum (VI). *Talanta.* **1996**;*43*:943-950.

5. Ananthi A, Kumar T.N, Mathiyarasu J, Joseph J, Phani K, Yegnaraman V. A novel potentiometric hydrogen peroxide sensor based on pKa changes of vinylphenylboronic acid membranes. *Mater. Lett.* **2011**;*65*:3563-3565.

6. Deyhimi F, Nami F. Modeling and optimization of the bi-substrate peroxidase-enzyme catalyzed potentiometric assay of hydrogen peroxide by response surface methodology with a central composite rotatable design. *J. Mol. Catal. B Enzym.* **2011**;*68*:162-167.

7. Jin G, Du S, Hu X. The petentiometric determination of peroxide hydrogen and glucose on the glassy electrode modified by the calix [4] arene. *Talanta.* **2009**;*80*:858-863.

8. Launer H.F. Determination of the pH Value of Papers. *J. Res. Natl. Bur. Stand.* **1939**;*22*:553-564.

9. Shamsipur M, Tashkhourian J, Hemmateenejad B, Sharghi H. Application of artificial neural network to simultaneous potentiometric determination of silver (I), mercury (II) and copper (II) ions by an unmodified carbon paste electrode. *Talanta.* **2004**;*64*:590-596.

10. Hu X, Leng Z. Highly selective and super-Nernstian potentiometry for determination of Cu^{2+} using carbon paste electrode. *Anal. Lett.* **1995**;*28*:979-989.
